# “They go hand in hand”: a patient-oriented, qualitative descriptive study on the interconnectedness between chronic health and mental health conditions in transition-age youth

**DOI:** 10.1186/s12913-022-09002-1

**Published:** 2023-01-02

**Authors:** Brooke Allemang, Susan Samuel, Karina Pintson, Megan Patton, Katelyn Greer, Marcela Farias, Keighley Schofield, Kathleen C. Sitter, Scott B. Patten, Andrew S. Mackie, Gina Dimitropoulos

**Affiliations:** 1 Faculty of Social Work, University of Calgary, MacKimmie Tower, 400-B3, 2500 University Drive, NW, Calgary, AB T2N 1N4 Canada; 2 Department of Pediatrics, Cumming School of Medicine, University of Calgary, 3300 Hospital Drive NW, Calgary, AB T2N 4N1 Canada; 3 Mathison Centre for Mental Health Research & Education, University of Calgary, 3280 Hospital Drive NW, Calgary, AB T2N 4Z6 Canada; 4 Department of Psychiatry, Cumming School of Medicine, University of Calgary, 3300 Hospital Drive NW, Calgary, AB T2N 4N1 Canada; 5 Department of Pediatrics, Stollery Children’s Hospital, 84400 112 Street NW, Edmonton, AB T6G 2B7 Canada

**Keywords:** Transition to adult care, Transition readiness, Youth, Chronic health condition, Mental health, Qualitative

## Abstract

**Background:**

Transition-age youth (TAY) with chronic health conditions frequently experience co-occurring mental health conditions. However, little is known about the perspectives of TAY with co-occurring diagnoses preparing to exit pediatric health and mental health services. Research is needed to understand the impact of a mental health condition on transition readiness and self-management in TAY with chronic health conditions.

**Methods:**

TAY (aged 16–20 years) with co-occurring chronic health and mental health conditions were recruited in Alberta, Canada. Nine semi-structured individual interviews were completed by phone or videoconference, and transcribed verbatim. Guided by qualitative description, we analyzed the data using thematic analysis in partnership with five young adults with lived experience in the health/mental health systems.

**Results:**

Participants shared their experiences living with simultaneous physical and mental health concerns and preparing for transition to adult care. Our analysis revealed three overarching themes: 1) “they’re intertwined”: connections between chronic health and mental health conditions in TAY, 2) impact of mental health on transition readiness and self-management, and 3) recommendations for service provision from the perspectives of TAY.

**Conclusions:**

Our findings highlighted the myriad ways in which physical and mental health are connected as TAY prepare for service transitions using specific examples and powerful metaphors. TAY endorsed the importance of providers discussing these connections in routine clinical care. Future research should involve co-designing and evaluating educational material addressing this topic with diverse TAY, caregivers, and service providers.

**Supplementary Information:**

The online version contains supplementary material available at 10.1186/s12913-022-09002-1.

## Background

The lifetime prevalence of depression and anxiety among Canadian youth aged 15 to 24 years are 11% and 12%, respectively [1, 2]. However, rates of mental health diagnoses (e.g., depression, anxiety) are two to three times higher in children and youth with chronic health conditions (e.g., asthma, cystic fibrosis, type 1 diabetes) compared to the general population [3–6]. In fact, between 20–59% of youth with chronic conditions also experience mental health conditions [7–9]. More severe physical health conditions and a higher number of underlying medical conditions are associated with greater functional impairment due to mental illness [10, 11]. While we recognize mental health conditions are health conditions, for the purposes of this paper, we define chronic health conditions as pediatric-onset physical health conditions which impact daily functioning for at least three months [12] and mental health conditions as brain-based medical conditions affecting mood, thinking, or behaviour [13]. Further, this distinction is important given the potential for fragmented service delivery within the health and mental health systems as youth age out of pediatric services.

Mental health conditions in youth with chronic health conditions are associated with decreased medication adherence, increased health care utilization, diminished school performance and poor quality of life [6, 14–17]. Individuals with co-occurring chronic health and mental health conditions experience prolonged hospitalizations, higher morbidity, and substantially higher health care costs than those with chronic health conditions alone [3, 17–19]. The presence of mental health comorbidities in youth with chronic health conditions may uniquely impact those in the developmental period of emerging adulthood, which corresponds with the desire for autonomy, simultaneous life transitions, and evolving family member roles [20, 21]. Mental health conditions may affect youths’ ability to take responsibility for their health during a period in which expectations for self-management are customary [3, 6]. Problematically, the effects of mental health conditions on chronic disease self-management in emerging adulthood specifically have not been well studied.

There are several barriers associated with the transition from pediatric to adult health and mental health care which occurs during emerging adulthood, contributing to increased health risks for youth with chronic conditions [22]. These include fear of leaving familiar pediatric providers, changes to parental involvement in care, a lack of specialized adult providers with knowledge of pediatric-onset conditions, and poor preparation for the adult system [22–26]. Youth with both health and mental health diagnoses may be leaving multiple pediatric clinics simultaneously, making care coordination and adaptation to adult services particularly challenging [27]. Differences in the types of services offered in adult care, including psychosocial support or a lack of a multidisciplinary approach, can also compound difficulties associated with transfer [28, 29].

A known facilitator of transition success in youth with chronic conditions is the development of health-related knowledge, decision-making, advocacy, and self-management skills, comprehensively referred to as ‘transition readiness’ [25, 29, 30]. While considerable research has focused on the concept of transition readiness, little is known about how self-management skills are developed and solidified depending upon the presence of a mental health comorbidity. In addition, there have been calls to further amplify the voices and perspectives of youth themselves surrounding pediatric-adult transitions to ensure their priorities are reflected in practice [31–33]. As such, research is needed to explore the transition readiness and experiences of transition-age youth (TAY) with co-occurring health and mental health conditions exiting pediatric services using a patient-oriented research (POR) approach.

### Objective

Our primary objective was to ascertain the experiences of TAY with co-occurring chronic health and mental health conditions exiting pediatric services using a POR approach. Specifically, we aimed to understand how readiness for transition from pediatric services is influenced by experiences of physical and mental health challenges in TAY.

## Methods

### Study design & setting

This patient-oriented, qualitative study was informed by the social-ecological model for adolescent and young adult readiness for transition (SMART) [34] and positive youth development [35], and conducted within the pragmatic paradigm [36, 37]. The SMART model posits that transition readiness for youth with chronic conditions is shaped by a series of factors, including those at the individual, family, community, and systemic level [34]. Within this model, youths’ age, health-related knowledge, and self-care skills are not commensurate with positive outcomes post-transfer to adult care [34]. Instead, the interplay between the aforementioned factors (and others, including the presence of a mental health comorbidity) should be considered by health care providers. Positive youth development asserts that young people’s competencies be leveraged in research and program design given they have the capacity to directly shape their own development [35]. Both theories influenced the conceptualization, analytic, and interpretation phases of the study, including the decision to conduct a patient-oriented, qualitative study. Pragmatism focuses on addressing social problems through inquiry and allows for the use of different types of data within a single study [38]. Our theoretical framework and pragmatic epistemological orientation positioned this study to be conducted using a POR approach to centre young adult voices in the project’s operation.

**Fig. 1:**
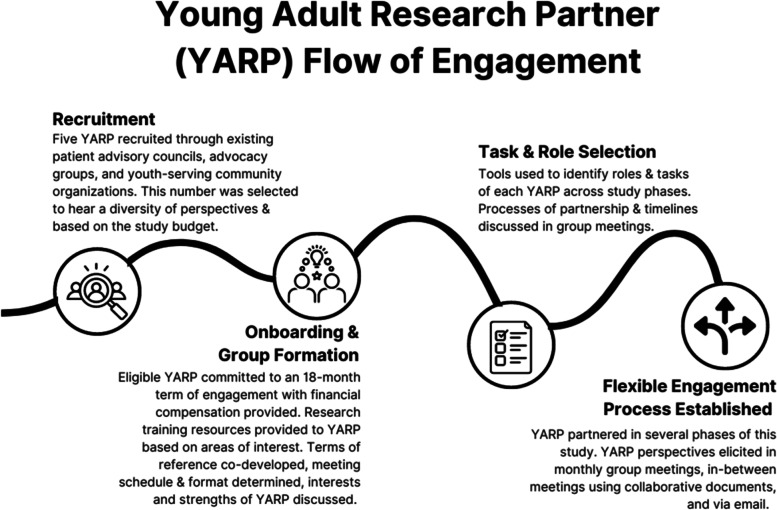
Young Adult Research Partner (YARP) flow of engagement

POR engages individuals with lived experience in the design, governance, and execution of research with the aim of producing data that is relevant to the communities under investigation [39]. This project was conducted in collaboration with a group of five young adult research partners (YARP) to bring the critical perspectives of young adults to the study design, to ensure the data was gathered in a youth-friendly manner, and to support the meaningful dissemination of our findings. The flow of engagement with the YARP, from recruitment to task selection, is outlined in Fig. 1. Eligible YARP were between 18–30 years old, identified as having either a chronic health condition, a mental health condition, or both, and resided in Canada. This age range was selected so that the YARP could reflect on and contribute their perspectives on previously experienced service transitions. See Supplementary Materials: Appendix 1 for the terms of reference document co-developed with the YARP at the outset of the partnership. Table 1 outlines the specific roles, tasks, and processes of partnership based on each stage of the research. The YARP were compensated for their time by way of monthly honoraria in line with the Alberta Strategy for Patient-Oriented Research SUPPORT Unit’s guidelines [40].

**Table 1:** Young Adult Research Partner (YARP) roles and processes of partnership by study phase

Study Phase	Young Adult Research Partner Role(s)^a^	Processes of Partnership
Recruitment	***Advisor:*** gives advice	YARP reviewed all email drafts and recruitment flyers developed by the first author to ensure materials were clear, written in accessible and youth-friendly language, and used appealing visuals.
	***Partner:*** works as an equal partner	Additionally, the YARP developed their own recruitment flyers and social media content (i.e., engaging videos shared on TikTok and Instagram) to share with prospective participants as these platforms would reach our target age group. Lastly, the YARP shared study information with individuals within their networks and community organizations to facilitate recruitment.
Interview guide development	***Partner:*** works as an equal partner	At the request of the YARP, a draft interview guide was written by the first author with a series of headings (e.g., background, intersections between health and mental health, transition readiness). The YARP reviewed the draft and elected to meet several times over a one-month period without the first author present to brainstorm and share ideas about each section of the guide. Next, the YARP assigned each partner to a different section of the guide and created a collaborative document that could be updated in real-time. The YARP were each responsible for reviewing their own section, adjusting the language of existing questions to enhance the guide’s youth-friendliness, and using their lived experience to compose their own relevant, open-ended questions. The YARP and first author then met, and each partner shared their edits, suggestions, and ideas about the guide with the group. The final interview was agreed upon by the first author and the YARP
Data collection	***Listener:*** is given information	The first author was responsible for conducting the interviews. Reflections about the interviews, the richness of the data, preliminary concepts arising in the interviews, and the processes of data collection were shared with YARP in monthly video-conference team meetings by the first author. Reflexive discussions focused on positionality, biases, assumptions, and values were held amongst the group
Data analysis	***Co-thinker:*** is asked to give opinion	The first author reviewed all interview transcripts and created an initial version of the codebook. The YARP were given the option to individually review interview transcripts and the codebook, highlighting the concepts, ideas, and codes they each felt were most pertinent to present in this article. Next, in a video-conference team meeting facilitated by the first author, the YARP came together to share their perspectives on which codes and subsequent themes should be included in this article. Their opinions on impactful codes and quotes were presented either verbally, or in written form (using shared documents including Google Jamboard, Google Docs) based on their individual preferences. Following a review of each YARP’s perceptions of the most prominent concepts, the first author and the YARP reached consensus about the final codes/themes by way of two video-conference group discussions, allowing the YARP to share their opinions on the language, final tables/figures, and illustrative quotes presented in this article.
Knowledge translation	***Partner:*** works as an equal partner	The YARP were involved in the preparation of conference presentation materials, delivered co-presentations at academic conferences and community events, and contributed to the preparation of this article.
	***Decision-maker:*** takes initiative, (final) decision	The YARP worked individually and collaboratively to develop and share social media content related to this study on our project specific TikTok and Instagram accounts. Accordingly, they took initiative to creatively disseminate the findings of this study and our partnership to relevant stakeholders (e.g., youth, caregivers, clinicians, researchers) and made final decisions about the content and method(s) of delivery.

^a^Roles adapted from Smits et al.’s Involvement Matrix [41] outlining the continuum of patient and public involvement in research

A qualitative description design was used, as this methodology aims to provide rich descriptions of a phenomenon that has not been thoroughly investigated in the existing literature [42–45]. We conducted this study in the province of Alberta, Canada, where there are three pediatric tertiary care hospitals serving large geographic regions, including urban and rural areas. Ethical approval for this research was obtained from the University of Calgary Conjoint Health Research Ethics Board (REB20-1928) and reporting of the results adheres to the Consolidated Criteria for Reporting Qualitative Studies [46].

### Participants & sampling

Eligible interview participants were English-speaking youth between the ages of 16–20 years who were diagnosed with at least one chronic health condition (e.g., diabetes, congenital heart disease) and one mental health condition (e.g., depression, anxiety) and resided in the province of Alberta, Canada at the time of the study. We elected to interview TAY aged 16–20 years old to elicit varying perspectives on transition readiness, given individuals in this age range would be at different stages of health care transition (e.g., preparing for transfer, post-transfer to adult care). Participants were recruited using purposive sampling techniques [47], allowing for the selection of TAY with relevant experiences in the health and mental health systems of care to address our research objective. Recruitment flyers co-designed by the YARP were distributed widely using social media (i.e., Instagram, TikTok) and by email to reach relevant clinicians, youth and family advisory councils, youth-serving community organizations, and post-secondary accessibility offices. The YARP also shared study materials within their networks. Interested participants were invited to email the research team for further details about the study. Study information was provided via email and reviewed by phone or videoconference prior to the interview. Signed consent was obtained for all participants. Our target sample size a priori was 10–15 participants based on existing health research using qualitative description [48, 49]. However, we remained flexible with our sample size, prioritizing the concept of information power, or the amount of information within the sample relevant to study the aim, within our data [50]. Of the 13 TAY who initially expressed an interest in this study, four did not respond to our efforts to schedule an interview. Data analysis and collection occurred simultaneously, thus, recruitment efforts ceased once we achieved information power, when no new concepts were emerging from additional interviews and our research question had been sufficiently answered by our participants [50].

### Data collection & analysis

Individual interviews lasting 45–60 min were conducted by phone or videoconference by the first author (BA) using a semi-structured interview guide (see Supplementary Materials: Appendix 2). BA is a female registered social worker and doctoral candidate, with clinical experience supporting TAY with chronic health conditions. The interview guide was developed in partnership with the YARP, focusing on relationships between health and mental health, preparing for transition to adult care, and self-management. Participants received the interview questions by email in advance of their interview.

The interviews were audio recorded, transcribed verbatim and all identifying information was removed from the transcripts to protect participants’ anonymity. Data collection and analysis occurred simultaneously in an iterative manner using NVivo software Version 12 [51]. A qualitative description approach [44] using thematic analysis [52] was used. Specifically, several authors (BA, KP, MP, KG) read and re-read the interview transcripts to make sense of the data. Each interview was then reviewed line-by-line by the first author, with codes being assigned to each meaning unit or piece of data. Where possible, codes were named verbatim using the language of the participants. Accordingly, a codebook was developed in an inductive fashion, with codes emerging directly from the interview transcripts [53]. The codes were then discussed amongst the research team, grouped, and categorized into broader themes and subthemes [52]. Overarching themes were agreed upon by all authors, including the YARP; thus, the YARP played a central role in the organization of the codes and the articulation of the final themes based on their lived expertise.

We adopted several strategies to ensure rigour and trustworthiness of the data. The interview guide was developed with feedback from a series of content experts from varying backgrounds, including social work, psychiatry, medicine, and lived expertise. By partnering with the YARP in designing the interview guide, we ensured the questions were youth-friendly and focused on pertinent issues to young adults themselves [39]. We recruited participants with different diagnoses to obtain a variety of perspectives regarding service transitions and to compare experiences across demographically diverse cases [54]. We used written memos throughout data collection and analysis to document our impressions of the data and held regular Zoom meetings to reflect on and discuss our biases, assumptions, and social locations in reference to the data [55]. We used thick description [56] by including direct quotations from participants throughout the results section. We used triangulation techniques [57] during data analysis by involving young adults with lived experience (i.e., YARP) in reflexive discussions about emerging codes and themes. These discussions served as an additional data source in our study, enhancing the trustworthiness of the findings [58, 59].

## Results

Nine participants were interviewed between February and June 2022. Participants ranged in age from 16 to 20 years old and identified as having a wide range of chronic health and mental health conditions. Demographic and clinical characteristics of the participants are presented in Tables 2 and 3, respectively. Three overarching themes were interpreted from the data in collaboration with the YARP: 1) “they’re intertwined”: connections between chronic health and mental health conditions in TAY, 2) impact of mental health on transition readiness and self-management, and 3) recommendations for service provision from the perspectives of TAY.

**Table 2:** Demographic characteristics of interview participants (*N* = 9)

Demographic Characteristics	n
Gender	
Female	5
Male	2
Non-binary	2
Age at time of interview	
16 years	1
17 years	2
18 years	2
19 years	1
20 years	3
Race	
Asian	1
Black	1
Middle Eastern	1
White/Caucasian	6
Immigration status	
Immigrant	2
Non-immigrant	7
Vocational status	
Employed (full or part-time)	2
High school student	3
Post-secondary student	3
Not currently in school/employed	1

**Table 3:** Clinical characteristics of interview participants (*N* = 9)

Clinical Characteristics	n
Chronic health condition category^a^	
Cardiovascular	1
Endocrine	1
Gastrointestinal	2
Genetic	2
Neurological	1
Renal	1
Respiratory	6
Rheumatological	2
Other	1
Mental health condition^b^	
Anxiety	8
Attention deficit hyperactivity disorder	2
Borderline personality disorder	1
Depression	6
Obsessive compulsive disorder	1
Phobia(s)	2
Post-traumatic stress disorder	2
Somatization	1
Age at primary diagnosis	
Birth to 10 years	6
11–14 years	3

^a^Participants could indicate more than one chronic health condition. A total of five participants reported having > 1 chronic health conditions

^b^Participants could indicate more than one mental health condition. A total of eight participants reported having > 1 mental health conditions

### “They’re intertwined”: Connections between chronic health and mental health conditions in TAY

There were a variety of ways TAY expressed connections between their chronic health conditions and their mental health throughout adolescence and emerging adulthood.

#### Negative emotions tethered to coping with chronic conditions

TAY shared how living with chronic health conditions frequently resulted in negative feelings, including grief, anger, worthlessness, worry, and hopelessness. Participants shared how these emotions directly contributed to the development or worsening of mental health symptoms:

*[Growing up with chronic kidney disease], I realized that I wasn't normal. Prior to my kidney transplant, I was like, ‘I will never grow up to be anything of value.’ I felt as though, mentally, I was going to go nowhere. I felt trapped in my own body, both literally and metaphorically, and that impacted my mental health negatively.* (109)

Others reflected on how they felt their illnesses increased their susceptibility to mental health challenges, *“the chronic conditions that I went through definitely put me in a more vulnerable position where the mental health issues came a lot more easily and more intensely than they might have for some other people”* (101). The stresses of living with severe, activity-limiting chronic health conditions with unpredictable trajectories created anguish for TAY:

*My major depressive disorder [was brought on by] the disease. I was scared something might happen, maybe I won’t reach the age of 30 because I've seen many young kids or many young adults dying and it gave me a lot of stress. I'm really scared most of the time. Any time I have a complication, I [fear] that maybe this is my last time.* (105)

In some cases, the debilitating effects of their chronic health conditions on their bodies and minds resulted participants wanting to *“give up”* (105). This was powerfully captured by one participant with severe asthma when he shared, *“when you're so tired after having to struggle to breathe, you're just like, I don't wanna do this anymore”* (102).

Lastly, the social aspects of living with a chronic health condition arose as a contributing factor to feelings of loneliness and isolation among TAY. Several participants experienced a lack of understanding from peers: *“you can feel really like you stand out, especially as a high school kid and are very different. You feel isolated and sometimes lonely because no one truly understands what's happening”* (103). They also described feeling *“behind everyone else”* (108) because of their conditions and *“miss[ing] out a lot on the social aspect of interacting with peers*” (101). One participant specifically outlined how her mental health was impacted by behaviours from her peers: *“healthcare providers don’t realize how people in school treat you differently or are mean because you have a chronic health condition, and the mental health effects that it has”* (103). Finally, flare ups and physical symptoms caused TAY to feel like they *“weren’t normal or capable of the things [they] wanted to do*” (101) in emerging adulthood.

#### Mental health effects of medications, procedures, & appointments

There were a series of connections between TAYs’ mental health and the treatments and routines they were expected to adhere to for chronic condition management. For instance, participants shared the mental health side effects from the medications prescribed for their chronic conditions, including *“anxiety, depression, or crazy nightmares*” (103) and *“feeling more sluggish and tired”* (107). One TAY also highlighted the implications of being on strong steroids for her lupus, namely, *“withdrawal, addiction, and mental health issues*” (103). Participants identified that attending hospital visits and interacting with a series of unfamiliar medical providers throughout childhood for reasons they could not understand impacted their affect. A TAY with cerebral palsy voiced that the multitude of assessments, procedures, and doctors’ appointments she attended as a child caused her to feel *“so nervous”* (106) that she was referred to a therapist to address the anxiety. In another instance, the surgeries and medical procedures required to address a TAY’s heart condition resulted in a diagnosis of medical post-traumatic stress disorder (PTSD):

*My mental health journey started about seven months after I had my heart surgery when I experienced my first PTSD flashback to a specific moment in surgery. The PTSD [subsequently] caused a lot of my generalized anxiety which was around having health conditions and doctor’s appointments and some of the treatments that go along with that.* (103)

#### The “vicious cycle”

While the mechanisms appeared to vary based on diagnoses, the compounding effect of physical and mental health concerns on TAY with co-occurring conditions was a commonly experienced phenomenon amongst participants. A TAY with Crohn’s disease, for example, described how her mental health impacted the severity of her physical symptoms: *“I think the amount of stress that I was experiencing due to my mental illnesses just made my Crohn's so much worse and my stomach hurt a lot more because of that big mind gut connection”* (101). Another participant highlighted the interactions between lupus and anxiety:

*I find that having both lupus and my anxiety and mental health stuff - it's like a vicious cycle. I'll have the anxiety and then I'll get the flare and then the flare causes more anxiety which makes the flare worse, in turn. It gets wrapped up in this whirlwind if there's not help and support to help me calm down some of the areas.* (103)

In one poignant example, a participant with asthma recounted an experience surrounding a hospital admission:

*I was under so much stress that my lungs wanted to stop working and would not go back to breathing right. I had to go into the intensive care unit because of the stress on my lungs because I was being bullied so harshly. It still follows me to this day… like if I get too stressed at work or if I'm really depressed, my asthma will kick start up and it's nasty.* (102)

These examples illustrate the cumulative and intersecting nature of physical and mental health concerns among TAY with a variety of co-occurring diagnoses.

#### The need for balance

The presence of co-occurring chronic health and mental health conditions amongst TAY necessitates self-awareness of how the two are related. Participants articulated a series of metaphors and strategies for balancing and attending to both their physical and mental health. One TAY used the analogy of a rock tower to describe this concept:

*There's definitely a connection. They all come together, and they all have to be in balance. Have you ever seen a cairn, like a rock tower? How it builds and builds? Sometimes it leans one way, and it leans the other way, and it can go all different directions. That is what I like to think of as an analogy when I think of my mental and my physical health. Because it can waver here and there and be okay. But if one thing comes down too much or is too heavy, everything falls over. (107)*

Participants highlighted how proper exercise, nourishment, sleep, and social supports benefitted their physical and mental health, stating the two could not be separated from one another, *“it’s practically the same, my physical and mental health”* (106). They advocated for medical professionals giving equal weight to physical and mental health to strike that delicate balance, and not neglecting one aspect over the other. As described by one TAY, *“[I wish health care providers] could balance both physical and mental health, put them on the same level, hand in hand”* (102). The notion of ensuring that both physical and mental health were viewed as priorities in their day-to-day lives arose in several interviews, as they felt avoidance of symptoms would lead to further problems. This was captured using another metaphor:

*I've been so much more healthy, and active, and living my best, full life ever since I realized I couldn't just prioritize one thing over the other. That I couldn't say my mental health is more important than my physical or vice versa. It's like two burning fires, right? If you put all your focus on one burning fire, the other one is going to go and destroy an entire forest. You got to keep both of them under control and [be sure] both of them are priorities.* (107)

In summary, participants recognized how their physical and mental health interacted, took action to address both aspects to create harmony and suggest that these connections be discussed more frequently among this age group.

### Impact of mental health on transition readiness & self-management

Participants shared their experiences coping with simultaneous physical and mental health concerns during a developmental period in which they were expected to begin taking more responsibility for their health. As such, two subthemes arose regarding the effect of TAYs’ mental health conditions on their ability to self-manage their chronic condition(s) and their level of preparedness for transition to adult care.

#### The effect of mental health on self-management

Participants described the many ways their physical and mental health were intertwined and affected how they coped with their chronic health conditions. Broadly, we heard from participants about the negative impact of their mental health on hygiene and self-care. As stated by one participant, “*my mental health was so bad that I did not want to take my pills in the morning, I barely left my room, I didn't shower, I barely ate, and I never wanted to talk to anybody*” (109). Inconsistent eating habits, for example, were interconnected with participants’ physical health and symptomatology: “*I had a bit of a close call when I hadn't been really taking care of myself all that well physically. I was skipping meals because I was in pain, and I just didn't feel like eating”* (107). Some participants expressed a “*diminished desire to keep going*” (102), and feelings of wanting to “*give up*” due to “*a lack of hope*” (105) following diagnosis. Of note, none of the TAY described suicidality explicitly, though two individuals alluded to prior instances of self-harming behaviours. The thought of having to adhere to strict treatment regimens “*to control some illness I didn’t choose to have for my whole life*” (105) felt unimaginable to the TAY interviewed. This, in turn, affected their capacity for self-management which included attending appointments, taking medications, and carrying out activities of daily living.

The most commonly reported effect of mental health on self-care among participants related to medication management. Many participants shared examples of how depression, for instance, impacted their adherence to treatment, “…*depression gets so intense you don't really care about yourself or care to look after yourself. In those moments, I would purposefully not take my medication because I knew that was just going to make me feel worse*” (101). Other participants outlined how specific mental health symptoms affected their medication routines. As stated by a TAY:

*With my asthma and my medication, a lot of the time, because of my ADHD, I'll forget to do my inhaler. Or if I'm depressed and I just can't bring myself to, or if I'm too tired, or if I'm falling behind in the morning, or if I slept in…there are a lot of different reasons. It's really difficult for me to take my inhaler.* (108)

An additional example of the impact of their mental health conditions on self-management surrounded appointment attendance. As highlighted by one participant:

*Even going to get medication or going to my doctors' appointments was made a lot more difficult with the anxiety. The anxiety made it very difficult for me to get on public transit and that actual realistic aspect of getting to my appointments was already made a lot more difficult with my social anxiety.* (101)

These concrete instances demonstrate the challenges TAY faced in caring for themselves and managing their chronic health conditions during emerging adulthood in the face of, often impairing, mental health symptoms.

#### The effect of mental health on transition readiness

**Fig. 2:**
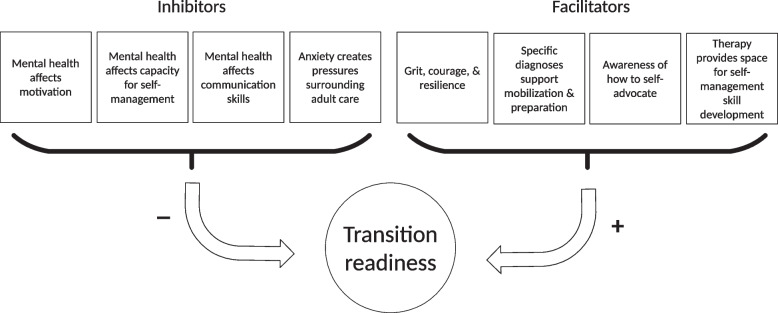
Impact of mental health conditions on transition readiness in transition-age youth with chronic health conditions

When asked to describe how their mental health influenced feelings of preparedness for transition to adult health and mental health services, participants shared a variety of perspectives. They reflected on the ways in which their mental health experiences served to both enhance and impede upon their transition readiness (See Fig. 2).

##### Mental health facilitates readiness

 Participants endorsed “*building up courage*” (105) for transition because of their interactions with multiple providers due to living with co-existing chronic health and mental health conditions. They also noted the ways in which mental health challenges allowed them to develop resilience and confidence in adjusting to new systems of care. As one TAY stated:

*[My mental health conditions] certainly helped me develop a lot more grit and an ability to adjust to new situations. I felt a lot more comfortable within that transition experience because I had already been exposed to such a diverse array of problems. I felt like, ‘whatever comes my way, I'll deal with it when it comes’.* (101)

Another participant highlighted how coping with multiple health and mental health conditions enhanced her readiness for transition specifically, sharing:

*One extreme benefit that I will always appreciate is the way [my conditions] have made me resilient and prepared to handle things. We don’t want our chronic conditions to get in the way of our dreams. So, we learn what we can, and we make those connections and those relationships [with providers]. And by getting all of that information, we get to make this [transition] plan.* (107)

An awareness of how to advocate for oneself was also described as a facilitator in the context of service transitions, *“I’ve really learned to advocate for my mental health needs, so that might be one of the reasons my transition readiness seems like a little bit more”* (103). Lastly, participants reflected on how their mental health conditions “*forced [them] to become more introspective and explore [their] emotional maturity*” (101) which, they felt, allowed them to consider how their mental health impacted their readiness for transition during emerging adulthood.

Another concept that arose from participant narratives was the perceived positive impact of specific mental health conditions, namely anxiety and attention deficit hyperactivity disorder (ADHD), on levels of preparedness for adult health and mental health care. They voiced feeling more ready for transition because of their tendency to overprepare for unfamiliar situations. TAY gave examples of how their mental health conditions motivated them to plan ahead by developing “*to-do lists”* (106), mapping out transit routes, and “*writing down questions*” (103) prior to interactions with new providers. One TAY aptly outlined her strategy for preparing for clinic appointments:

*I try to give an hour between when my appointment is, and an hour after to make sure I'm oriented, not worried, and not anxious. Because I can get stuck in one place, and I'll just crawl into a little sadness because it's overwhelming, and I hate being overwhelmed.* (106)

Many participants voiced how anxiety led to feelings of mobilization:

*I don't wanna be left in the dark and not knowing what's gonna happen with my health. I feel like my anxiety is great for when I do have [asthma] attacks or things happen. Cause I'm like, ‘I need to figure this right now because something's gonna happen’*. (102)

Lastly, some participants shared how their mental health providers, including therapists and psychologists, worked with them on developing coping strategies, working towards independence, and preparing for transition to adult health services. These positive experiences in counselling appeared to bolster TAYs’ capacities, allowing them to feel equipped to manage their health care transitions with greater confidence. For example:

*I’ve been doing a lot of work with my psychologist for the mental health part and how I can transition and gain more independence during appointments where I feel really comfortable because I’ve always gone into all of my appointments with my mom...so she has been transitioning me to do it on my own. [Those skills] can help with some of my other stressful healthcare appointments too.* (103)

Of note, several TAY interviewed did not have access to consistent mental health support.

##### Mental health impedes readiness

While mental health conditions were found to promote readiness among many participants, we also heard about how these diagnoses contributed to challenges preparing for health and mental health service transitions. Some young adults voiced *“feeling stuck”* as a result of their mental health, and lacking the motivation to keep track of appointments, file taxes, and *“do the things [they are] supposed to do”* (108) to become the managers of their own health care. Participants recognized the episodic nature of their mental health symptoms, noting that on “*bad days*” they would be mentally (and physically) unable to carry out tasks like “*calling in a prescription*” (102), for example. TAY reflected on other instances in which their mental health impacted their capacity to remember appointments, plan for clinic visits, or write down questions for providers due to “*depressive episodes*” (105) or ADHD causing things to be “*jumbled up in [their] mind*” (108). The effect of anxiety, in particular, on one’s ability to communicate confidently with health care providers was summarized by a participant, “*I get anxious with talking to people at doctor's appointments. Sometimes when I'm anxious, I'll go completely silent. I'm like, ‘I don't know what to say. Am I supposed to say something? Oh no.’ And then I just spiral”* (108). Participants also shared how their mental health conditions added pressure and resulted in feelings of overwhelm regarding upcoming transitions to adult specialty clinics. Having anxiety was described by one young adult as leading to “*disorientation*”, “*worry about what might possibly [go wrong]*” and “*discomfort*” (106) in getting ready for attending appointments independently in the adult system.

### Recommendations for service provision from the perspectives of TAY

Based on their experiences navigating the health and mental health systems during emerging adulthood, participants highlighted a series of recommendations for service providers working with TAY with co-occurring chronic health and mental health conditions. See Table 4 for actionable steps and additional participant quotations coinciding with each recommendation.

**Table 4:** Recommendations for service providers with supporting quotations

Recommendation	Actionable Steps	Supporting Quote(s)
Discuss connections between physical & mental health	Give equal weight to physical and mental health during clinic appointments	*“I feel it's important because in general, people don't really see mental health as something as important as physical health. But I think especially if people realize that they're so connected and intertwined, especially with people who do have both mental health issues, and physical health issues, that one affects the other. So I feel like they pay more attention to mental health issues.” (108)* *“I really do think that it is essential, that that is understood. Because for so many times, I felt like I was losing my mind. I thought I was going crazy and insane. And I wasn't able to make that connection because I was so young. Right? I wasn't able to make that connection that my physical health and my mental health, they went hand in hand. They go together. And it is important to make both of them a priority. I think having that information and giving it out to people would be so beneficial.” (107)*
	Ensure service providers/clinics have adequate knowledge in health & mental health to discuss connections	*“I'm not sure what training therapists, psychiatrists or mental health providers go through? And same with doctors who deal with physical illness, but having both parties knowledgeable in both areas. I know that's a lot to ask. Because there is so much to know. I know there's some family doctors who wouldn't even touch mental health medication because they don't know much on it.” (104)*
	Tailor services to the needs of TAY with co-occurring chronic health and mental health conditions using interprofessional teams and/or innovative clinical approaches (e.g., combined clinics)	*“I think you should have specific care for them, maybe a specific clinic where you can receive both. Yeah. It'll make it easier and not only easier for me and also easier for the doctor, because if you have all my history and the follow up even will be much easier.” (105)*
Offer strengths-based, validating care to TAY	Bolster TAYs’ capacities	*“Yes, it does suck, yes, my life is a lot harder than maybe some other kids my age, but I'm also still capable. And I already experience so much of, ‘oh, you poor thing’, and the ‘why me?’ type of mentality from my parents and from myself—that's not what I need. I need to be told that I'm capable of being able to stand up and [advocate for myself] and I wish that I saw that more from my healthcare providers.” (101)*
	Validate TAYs’ experiences	*“Believe the patient’s experience, the extent of their pain.” (101)* *“I think one of the big struggles, especially with working with mental health conditions in particular with minors is that the doctors there, they tend to migrate towards what the parent wants or needs, because the kid has a mental condition. Right? There's, I don't want to say a bias. But it could be just in the back of their mind, subconsciously they think this kid isn't able to make a choice for themself because of this condition. Which isn't the case. Sometimes the kid knows exactly what they need.” (107)* *“It's hard for someone to understand the pain you go through unless you're in that pain or unless you deal with that illness. But I feel like just honestly, just being kind of understanding because, I know I do complain about my hand a lot. And in the past my therapists would kind of just brush it off.” (104)*
	Speak directly to TAY in clinical encounters (with or without caregivers present)	*“Make sure for healthcare providers, when you're talking with someone who's coming up on transitioning, turn and look at them and ask them what they think, and what options they're considering, and what things they aren't comfortable with. Because if healthcare providers always turn to the parent, that kid is never going to be ready. That kid is going to get thrown into this very scary, new transition thing where the doctor’s going to ask them what want to do, because there's no parent in the room. And that kid is going to freeze up. That kid has never had to make a decision regarding their own health before.” (107)* *“I think also regularly, making it more normalized to send the parents out of the room every visit. Just in case the minor in question has anything that they want to bring up, or if they need some support and they can't tell their parents, or are too embarrassed for whichever reason. Because that was an issue a lot for me—I wanted to bring things up, but I couldn't.” (108)*
Make mental health services accessible to TAY with chronic health conditions	Bolster the availability of low-cost mental health services for TAY	*“I'm currently trying to get diagnosed with like autism and, and other mental health issues and that's been a struggle because it's intense in trying to find people and I'm also low income.” (102)* *“I think more resources on counseling for minors who can't pay for therapy, or their parents can't, or won't pay for therapy, or stuff like that. Just so they can get the mental health help that they need.” (108)*
	Ensure TAY have a point of contact during service transitions	*“I've accessed a lot of the healthcare system and I’ve fallen through the cracks and stuff like that. I'm with the gender program, which has a psychiatrist or a psychologist who does referrals and checks up on me every once in a while, but I'm trying to find someone who will be like, ‘Yes, I got you’.” (102)* *“One program was just like, ‘okay, bye’. And I'm just like, wait? I still need trauma therapy because I’m messed up, you know? And because it took me a long time to get that therapist that I have now.” (104)*
	Send adult mental health referrals early given wait times for services	*“I actually have the appointment [with an adult psychiatrist]. It's just a long wait until the actual appointment.” (104)*

#### Discuss connections between physical and mental health

Most participants described not having received sufficient information about connections between physical and mental health in either the pediatric or adult health care systems. As stated by one participant, “*I’m part of six different [specialty] clinics, but I haven’t had any of my doctors bring up any of those things. I feel like there's a huge disconnect*” (103). Some specialists, including gastroenterologists or community-based psychologists, outlined the “*mind-gut connection*” (101) or “*the bridge [between] chronic illnesses and mental health stuff*” (103), but even this information appeared to be limited. As such, participants endorsed the importance of having service providers treat them as a *“whole person”* (102) by discussing connections between physical and mental health in routine clinical care. TAY noted the value of emphasizing how one may impact the other, given “*they’re so connected and intertwined…you can’t help yourself if you don’t know what’s wrong*” (108). They advocated for embedding conversations about these connections into appointments to ensure young people understand these conditions can *“go hand in hand [because] it is important to make both [physical and mental health] a priority”* (107). Proactively describing possible mental health side effects of medications, asking how youth are doing during appointments, and acknowledging that living with a chronic condition can be *“hard and emotional”* (103) were some ways TAY felt this connection could be raised by providers. Participants advocated that “*[chronic health] clinics [receive] more mental health training so that they’re equipped to address some of these specific [interactions]”* (103). Lastly, TAY recommended that service delivery be tailored to the needs of the TAY with co-occurring diagnoses. They suggested, for instance, that equal weight be given to health and mental health concerns in clinical encounters, as individuals coping with simultaneous physical and mental health challenges may “*need different options*” (102). Other TAY felt that interprofessional teams or *“a specific clinic where you can receive both [health and mental health care]”* (105) be considered.

#### Offer strengths-based, validating care to TAY

Participants vocalized their desire to be viewed as capable of making decisions about their health and mental health care by service providers. They recommended clinicians aim to bolster their existing strengths by believing and validating their experiences and supporting them to work towards more independence in the context of their care. As shared by one TAY, *“healthcare providers giving the kid the chance to start learning that independence, I think it'll make it a lot easier for them and a lot less scary about this whole idea of transitioning”* (107). Some TAY felt dismissed or discredited by health care providers due to their mental health diagnoses. Thus, TAY suggested providers adopt a series of skills and approaches when working with those with co-occurring health and mental health conditions, including active listening, aiming to understand the youth’s perspective, and believing their symptoms (e.g., pain, anxiety) were real. Several participants endorsed the concept of “solo time” or having caregivers step out of the room to allow youth to raise questions or concerns they may not otherwise feel comfortable discussing with family members present. This was particularly relevant for TAY experiencing mental health issues when their *“parents weren’t super open about mental health stuff”* (108). Even when caregivers were present, TAY emphasized the criticality of having service providers direct their attention and questions to the youth to give them the opportunity to respond and gradually develop confidence in interactions with clinicians.

#### Make mental health services accessible to TAY with chronic health conditions

The accessibility of timely, developmentally appropriate mental health services for TAY with co-occurring health and mental health conditions around the time of transition was a key issue raised by participants. Long waitlists for publicly funded mental health services, changes to eligibility criteria after age 18, and the costs of private therapy were some of the barriers outlined by TAY with chronic health conditions who were already facing a series of simultaneous service transitions. Therefore, participants suggested the clinicians overseeing the medical care for their chronic health condition(s) (e.g., respirologists, gastroenterologists) consider referring TAY to free or low-cost services (where possible) early to ensure youth requiring mental health support were attached to a service provider before transitioning to adult care. They also advocated for ensuring the mental health providers TAY were referred to were knowledgeable about chronic health conditions, health care transitions, and the connections between physical and mental health during the developmental period of emerging adulthood, given the two are inextricably linked in this population. Given the long waitlists for mental health services experienced by participants, they recommended clinicians introduce the topic of transition early and begin identifying possible adult services (and referral pathways) before they turned 18. Several participants described the impending transition to adult health care as exacerbating their mental health symptoms which in turn impacted feelings of readiness for adult care. Finally, TAY advocated for ensuring they had a point of contact during transitions to adult services, given the myriad clinics they may be attending (e.g., specialists, primary care, mental health). Many participants described experiences of *“falling through the cracks”* (102) amidst a series of simultaneous transitions, and how helpful it would be to have a person they could connect with regarding questions, waitlists, and timelines.

## Discussion

This research sought to explore the experiences of TAY with co-occurring chronic health and mental health conditions exiting pediatric services. Our findings indicated the myriad ways that physical and mental health are connected during the developmental period of emerging adulthood, including the implications of co-occurring diagnoses on school performance, socialization, transition readiness, and self-management. TAY also highlighted a series of recommendations for service providers supporting youth during service transitions. Given this work was conducted in partnership with the YARP, our study demonstrated the value of collaborating with individuals with lived experience throughout the research process.

Minimal qualitative research has focused on the intersections between chronic health conditions and mental health, or the implications of these diagnoses on the adolescent population specifically. Our results regarding intersections between physical and mental health echo prior research examining the impact of mental health conditions on quality of life and well-being in youth with chronic conditions. For instance, previous research on the psychological aspects of chronic health conditions in children and adolescents indicates that the severity of symptoms experienced, the visibility of their physical illness, levels of pain, uncertainty of prognosis, and feelings of control over symptoms influence the psychological effect of chronic conditions [60]. Indeed, the TAY interviewed in this study described the debilitating effects of specific physical symptoms (e.g., lupus flares, asthma attacks, gastrointestinal symptoms) and the unpredictable trajectories of their illness course as negatively impacting their mental health. In their study, Barnes et al. [61] also identified that youth with co-occurring physical and mental health conditions had the lowest levels of emotional well-being compared to those with solely a physical health or mental health condition, and those without chronic conditions. This point speaks to the importance of the intersecting and compounding nature of simultaneous health and mental health conditions that was described by TAY using the metaphor of the ‘vicious cycle’ in this study. Lastly, the onset of chronic health conditions in childhood and adolescence has been found to increase the likelihood of major depressive disorder and anxiety disorders in emerging adulthood [9]. This was noted by our participants who described living with their chronic health conditions increased their susceptibility to, and/or precipitated the development of mental health conditions. 

To our knowledge, the implications of mental health comorbidities on transition readiness in TAY with chronic health conditions have not been well-studied, given most studies addressing this issue have only included samples of youth with singular (and not co-occurring) diagnoses [62–64]. The insights participants shared regarding how their mental health served to both enhance and impede their preparedness for transition contributes to a growing body of evidence on the topic of co-occurring diagnoses and readiness for transition, and suggests this area requires further research. The impact of a mental health condition on chronic disease self-management more broadly, however, has been well-investigated. Our results bring the TAY voice to previously identified associations between the presence of mental health comorbidities and problems with medication adherence [14, 60, 65]. They expand on our understanding of the factors and pathways that make it challenging to consistently adhere to a treatment regimen in the midst of a mental health episode among the TAY population. Our findings provide greater context to prior research indicating that individuals with depression/anxiety were less likely to attend outpatient appointments for their chronic condition [66]. The TAY interviewed in this study shared nuanced examples of how and why their mental health impacted their ability to attend appointments, confidently communicate with providers, and adjust to new systems of care following transfer to an adult provider. These narratives bring to light the unique challenges faced by TAY, specifically, given they are expected to self-advocate and take on responsibility for their care for often the first time.

The recommendations provided by participants in this study have direct implications for clinical practice and align with national practice guidelines for youth transitioning to adult care [67]. The concepts of introducing transition and initiating appropriate referrals early, identifying a point of contact or "transition champion” throughout the process, and gradually working to support autonomy were common themes among the interviews and the Canadian Paediatric Society’s recent position statement on transitions to adult care [67]. The importance of validating youth’s experiences and bolstering their capacities were emphasized by TAY in our study, suggesting the adoption of a resilience and strengths-based approach to transition preparation which has been previously described [68]. While specialized services exist to address psychiatric concerns in pediatric inpatients (e.g., consult-liaison psychiatry) [69], TAYs’ recommendation of discussing connections between chronic health conditions and mental health in outpatient specialty clinic appointments during emerging adulthood and implications for self-management appears to be a novel finding.

Finally, the collaboration with the YARP yielded lessons learned about partnering with young adults in research that have implications for future POR projects. Importantly, the YARP had autonomy over the tasks and methods of involvement in this research project. Each YARP made decisions about their desired contributions at different stages of the project based on their interests and personal goals. This flexibility was endorsed as an element of success within the group. The YARP voiced appreciation for having guidance on some aspects of the project from the first author, given many were new to research (e.g., a draft interview guide to work from), and for more independence on other aspects (e.g., informally brainstorming interview guide questions) to support their growth. The inclusion of the YARPs’ voices in designing the data collection tool, recruitment strategy, analysis, and interpretation of the findings ensured that the data and results were grounded in lived experiences and supported authentic decision making among partners throughout the project’s entirety. The interviews yielded rich, nuanced information that would not have been possible had the guide been developed by researchers alone. For instance, two YARP used their experiences as previous research participants to reword questions they felt would not prompt extensive responses. The feedback and questions posed by the YARP throughout data analysis and interpretation encouraged the research team to reflect on the data in new ways. For example, one partner shared the impact of having a therapist on her own transition readiness, and suggested we explore the role of mental health supports in promoting readiness amongst our participants. Opportunities for the YARP to share their own knowledge and expertise (e.g., regarding social media strategies) with the group served to enhance feelings of connection within the YARP, built capacity among partners, and allowed for reciprocal learning with researchers. This project enacted well-established components of successful youth-adult partnership frameworks, including flexibility, authentic decision-making, and reciprocal learning [70], demonstrating how to apply these in practice whilst simultaneously promoting growth amongst the YARP and researchers alike.

### Limitations

This research was not without its limitations. While efforts were made to recruit a socio-demographically diverse sample using our social media strategy and by sharing our goal of hearing from specific groups of youth when meeting with clinicians, the majority of our participants were white. The pandemic made recruitment challenging, as an in-person presence in clinics and diverse youth-serving agencies was not possible. Thus, we relied primarily on social media, emails, and virtual meetings with service providers to support recruitment which may have impacted the demographic of our sample. Further research exploring intersections between health and mental health conditions in TAY is needed among racialized, Indigenous, and LGBTQIA2S + youth, immigrants, and refugees to elucidate their experiences throughout transition to adult care. This research was conducted in the province of Alberta, Canada and most participants resided in urban or suburban communities. Thus, the findings may not be generalizable to other contexts (e.g., rural communities, international settings) where health services are delivered differently. While our sample size was small, we heard from TAY with a wide range of diagnoses, genders, ages, and vocational statuses which was a strength of this research. The engagement of the YARP throughout interview guide development, data analysis, and interpretation ensured the voices of young adults with lived experience were centered in the presentation of results. Importantly, the YARP consisted of a group of highly engaged individuals who had opportunities to interact with peers and reflect on how their chronic condition(s) influenced their identity development throughout the duration of this project. Social support for young people with chronic conditions transitioning to adulthood facilitates the integration and acceptance of their chronic conditions into their identities [71]. Thus, examining the impact of patient engagement opportunities on identity formation in young adults with chronic conditions, and the role of social support on transition readiness are important future directions in this field.

## Conclusions

Substantial research suggests that individuals with co-occurring chronic health and mental health conditions are a unique group, who may require tailored transitional intervention plans to meet their needs. To date, relationships between physical and mental health in TAY exiting pediatric services specifically have not been sufficiently investigated. This POR study provides evidence of the unique, rich, and nuanced experiences of this age group as they navigate service transitions and developmental milestones whilst coping with simultaneous health and mental health concerns. It provides practical recommendations for service providers and suggests that connections between physical and mental health be acknowledged and addressed in the clinical context. Future research in this area should explore the perspectives of underrepresented communities during service transitions, including racialized and LGBTQIA2S + youth, newcomers, and refugees. The experiences of caregivers and service providers regarding the needs of TAY with co-occurring health and mental health conditions should also be examined to provide a multifaceted view of this topic. Research focused on developing and evaluating educational interventions for both TAY and service providers regarding transitions to adult care and intersections between chronic health and mental health conditions could also be explored.

## Supplementary Information

**Additional file 1.**

**Additional file 2.**

## Data Availability

The datasets generated and/or analysed during the current study are not publicly available due to their ongoing analysis for preparation of a thesis dissertation but are available from the corresponding author on reasonable request.

## Abbreviations

LGBTQIA2S+: Lesbian, Gay, Bisexual, Transgender, Queer, Intersex, Asexual, Two-Spirit
POR: Patient-oriented research
SMART: Social-ecological model for adolescent and young adult readiness for transition
TAY: Transition-age youth
YARP: Young adult research partners
