# Supplementary material for: “They go hand in hand”: a patient-oriented, qualitative descriptive study on the interconnectedness between chronic health and mental health conditions in transition-age youth
Source: BMC Health Serv Res. 2023 Jan 2;23:2. doi: 10.1186/s12913-022-09002-1 (PMC9809059; doi:10.1186/s12913-022-09002-1)
Supplement: Supplementary file 1 — Additional file 1. [file 12913_2022_9002_MOESM1_ESM.docx]

**Supplementary Materials: Appendix 1**

**Terms of Reference – Young Adult Research Partners (YARP)**

**Background:**

The purpose of the **READY2Exit (**Readiness and Experiences of ADolescents and Young Adults with Co-Occurring Health and Mental Health Issues Exiting Pediatric Services) research study is to better understand how young adults who have health and mental health issues prepare for their transition out of pediatric services. We are especially interested in how young adults cope with their health and mental health, and how they feel health/mental health professionals can best support them during the transition period. We are also interested in what young adults feel influences their readiness for transition to adult services and the potential impact of the COVID-19 pandemic on their readiness. This study will be conducted in collaboration with the YARP over a two-year period to ensure the voices of young people are heard within the research.

**READY2Exit Objectives:**

The goals of this study are to:

1. determine whether mental health issues influence transition readiness in young adults with chronic health issues
2. understand the transition experiences of young adults with health and mental health issues as they prepare to leave pediatric services.

**YARP Role:**

Each YARP member will determine how they would like to contribute to the project based on their interests, skills & availability. Specific roles & tasks will be determined together during our first few meetings & can be revisited at different timepoints throughout READY2Exit.

YARP members will be invited to collaborate on different tasks, including:

- Interview guide development
- Determining how to communicate study results to youth, caregivers & health care providers
- Sharing study findings widely (i.e., co-presentations)

YARP input may be sought through a variety of methods, including feedback by email, one-on-one phone/Zoom calls, and group meetings by phone or Zoom. This engagement will take place from approximately March 2021 to March 2023 with the option to continue for longer.

These terms of engagement will be reviewed, revised and approved by all members of the YARP.

**Guiding Principles of the YARP:**

1. All members are to be treated with respect, and no member shall dismiss or seek to invalidate the experience of another member.
2. All members are to be recognized as experts in their own experience. No one can be wrong about what their experience is.
3. All personal information is to be treated with strict confidentiality within the group.
4. All members will have their voices heard, and the utmost effort will be taken to incorporate their advice into this study. Multiple forms of knowledge including experential, Indigenous, technical and academic will be recognized and valued. This principle will be evaluated with a post-study survey.

**Membership:**

The READY2Exit YARP will be comprised of up to five Canadian young adults (aged 18-30 years) with lived/living experience in the health and/or mental health systems. The YARP will collectively bring the perspective of young people with health and mental health issues to READY2Exit. Members will provide their own personal observations, advice and contributions and not those of any particular group to which they belong.

YARP members are committed to attend meetings (video or teleconference), review materials in advance of meetings, engage in meeting discussions (using verbal or written communication) and provide feedback using multiple forms depending upon their communication preferences.

**Engagement Options:**

Feedback will be sought by email, phone and/or Zoom meetings. The frequency and time of Zoom meetings will be determined in collaboration with YARP members. Meetings are expected to run for a maximum of 90 minutes each.

**Confidentiality:**

Participation in the YARP will be voluntary, though compensation will be offered. YARP members will be provided with resources to support onboarding/training. It will be possible for YARP members to withdraw from this project at any time without prejudice. The READY2Exit research team will encourage confidentiality of the discussions that occur during YARP meetings. The meetings may be recorded to ease note taking. YARP members will be given the option to be identified or remain anonymous when sharing study findings.

**Compensation:**

The YARP members will be compensated in accordance with the guidelines set out under the SPOR Evidence Alliance Patient Partner Appreciation Policy and Procedure. An hourly rate of $25 will be offered, with the anticipated time commitment of approximately 1-3 hours/month for 24 months.

<https://sporevidencealliance.ca/wp-content/uploads/2019/08/SPOR-EA_Patient-Partner-Appreciation-Policy-and-Procedure.pdf>

**Equity, Diversity and Inclusion:**

The READY2Exit team & YARP members are committed to modeling the equity, diversity and inclusion protocols as determined in the READY2Exit study protocol. Namely, individuals of diverse backgrounds are encouraged to participate in the research and contribute their lived experience to this project.

**Communication:**

YARP members are encouraged to contact Brooke Allemang (PhD Candidate on READY2Exit) with any questions, communication needs or accessibility concerns at [brooke.allemang1@ucalgary.ca](mailto:brooke.allemang1@ucalgary.ca)
