# Supplementary material for: “They go hand in hand”: a patient-oriented, qualitative descriptive study on the interconnectedness between chronic health and mental health conditions in transition-age youth
Source: BMC Health Serv Res. 2023 Jan 2;23:2. doi: 10.1186/s12913-022-09002-1 (PMC9809059; doi:10.1186/s12913-022-09002-1)
Supplement: Supplementary file 2 — Additional file 2. [file 12913_2022_9002_MOESM2_ESM.docx]

**Supplementary Materials: Appendix 2**

**Interview Guide**

***Background***

- Can you start by telling me a bit about yourself?
  - Probe asking to highlight in their own words- experiences with school, favourite subjects, hobbies/extracurricular activities, family life, friends, etc.
- So that we can get a sense of the backgrounds of our interview participants, I’m going to ask you for a few demographic details. Can you please tell me your gender? Ethnicity? Age? Whether you live in an urban or rural setting? Whether you or your parents immigrated to Canada?
- What interested/motivated you to participate in this study? Have you ever participated in a study like this before?
  - Probes: Were you hoping to learn more about yourself? Interested in furthering research about support for others?
- If you’re comfortable discussing, can you share with me a bit about your physical health condition?
  - Probes: What is/are your diagnosis/es? What does living with your condition mean for you? What impact does it have on your daily/overall life in your own words? How long have you lived with your condition?

***Mental health***

- Have you ever been diagnosed with a mental health issue/condition?
  - If yes, can you please share what you have been diagnosed with?
  - If no, would you self-identify as having a mental health issue? If so, which one(s)?
- Have you ever received professional help from a mental health practitioner or service?
  - If yes, what kind of support? What type of setting? Did the service meet your needs? How long did you receive mental health services for?
- Are you currently receiving support from a mental health professional?
  - If yes, what type of support? (i.e., community-based, hospital-based, virtual vs. in person)
  - Do you think you will continue to seek mental health support after you age out of pediatric services?
- Do you view anxiety/depressed/stressed feelings as a clinical diagnosis or as a part of your life?

***Intersection between mental and physical health***

- Can you tell me one specific story or instance when you felt your mental health impacted your physical health? Or vice versa?

Has your mental health ever gotten in the way of your ability to manage your chronic health condition? (i.e. take your medications, go to appointments)?

- Do you know if your (*specific clinic*) has a mental health professional on their team (i.e. psychologist, psychiatrist, social worker)?
  - If yes, have you met with them?
- Are you aware of what mental health services will be available in adult care (specialty clinic)?
- Did you get any information about how your physical and mental health are related?
  - If yes, was that information helpful?
- Do you feel that your health and mental health needs are being met currently?
  - Probes: If yes, where are they being met? Are they being met in one clinic or multiple?
- Do providers talk to you about how your physical and mental health are related to each other?
  - If not, how could these needs be better met? What do you think holistic care would look like for you?
- What suggestions do you have about how to improve services for youth (like you) with chronic conditions and mental health issues in Alberta?
  - This could be things like services being provided in a “one-stop shop”, community-based care or hospital-based care.
  - What professional backgrounds? Peer mentors/support? Groups vs. individual? Adolescent-specific care?
- How has the COVID-19 pandemic impacted how your health or mental health care is delivered?
  - Probes: Telephone or video-conferenced visits? Frequency of appointments? Emergency department use?
- How has the COVID-19 pandemic affected how you manage your health or mental health?
  - Probes: Have coping strategies changed at all (i.e., peer support, extra curricular activities)? Have you had to become more or less independent? Have the stay-at-home orders affected your health or mental health?

***Transitions & Readiness***

- Overall, how prepared do you (or did you) feel for the transition to adult services?
- Are there specific areas you feel more/less confident with relating to transition readiness? Why do you think that is?
  - Probes: Managing medications, communicating with care providers, etc.
- Are there ways that your mental health impacts your readiness for transition (in either positive or negative ways)? Can you tell me about why/why not?
- What is helping you prepare for transition?
- What role does your support system (i.e., family, peers, etc.) play in preparing for transition?
- How can health/mental health professionals best support you in transitioning to adult services?
  - Probes: What kinds of information/resources/emotional support do you need?
- What suggestions do you have about how to improve (health and/or mental health) service transitions for youth with chronic conditions and mental health issues in Alberta?
  - Probes: Early intervention? Services for 12-25 year olds? Joint pediatric-adult transition clinics? Youth-friendly resources? Peer support workers? Transition navigator?

***Knowledge Translation***

- How do you suggest sharing the results of this study with other adolescents with co-occurring health and mental health issues?
  - Different social media platforms? What kind of content (i.e., videos, infographics)?
- How do you suggest sharing the results with healthcare providers and policy makers (those who are responsible for creating ideas and plans for businesses and the government)?
